# Standardized Saponin Extract from Baiye No.1 Tea (*Camellia sinensis*) Flowers Induced S Phase Cell Cycle Arrest and Apoptosis via AKT-MDM2-p53 Signaling Pathway in Ovarian Cancer Cells

**DOI:** 10.3390/molecules25153515

**Published:** 2020-07-31

**Authors:** Youying Tu, Lianfu Chen, Ning Ren, Bo Li, Yuanyuan Wu, Gary O. Rankin, Yon Rojanasakul, Yaomin Wang, Yi Charlie Chen

**Affiliations:** 1 Department of Tea Science, Zhejiang University, Hangzhou 310058, China; youytu@zju.edu.cn (Y.T.); c.lianfu@foxmail.com (L.C.); ningren@zju.edu.cn (N.R.); drlib@zju.edu.cn (B.L.); yywu@zju.edu.cn (Y.W.); 2 College of Health, Science, Technology and Mathematics, Alderson Broaddus University, Philippi, WV 26416, USA; 3 Department of Biomedical Sciences, Joan C. Edwards School of Medicine, Marshall University, Huntington, WV 25755, USA; rankin@marshall.edu; 4 Department of Pharmaceutical Sciences and WVU Cancer Institute, West Virginia University, Morgantown, WV 26506, USA; yrojan@hsc.wvu.edu; 5 Key Laboratory of Horticulture Plant Biology, Ministry of Education, College of Horticulture & Forestry Sciences, Huazhong Agricultural University, Wuhan 430070, China

**Keywords:** tea (*Camellia sinensis*) flowers, BTFS, A2780/CP70 ovarian cancer cells, apoptosis, S phase cell cycle arrest

## Abstract

Ovarian cancer is considered to be one of the most serious malignant tumors in women. Natural compounds have been considered as important sources in the search for new anti-cancer agents. Saponins are characteristic components of tea (*Camellia sinensis*) flower and have various biological activities, including anti-tumor effects. In this study, a high purity standardized saponin extract, namely Baiye No.1 tea flower saponin (BTFS), which contained Floratheasaponin A and Floratheasaponin D, were isolated from tea (*Camellia sinensis* cv. Baiye 1) flowers by macroporous resin and preparative liquid chromatography. Then, the component and purity were detected by UPLC-Q-TOF/MS/MS. This high purity BTFS inhibited the proliferation of A2780/CP70 cancer cells dose-dependently, which is evidenced by the inhibition of cell viability, reduction of colony formation ability, and suppression of PCNA protein expression. Further research found BTFS induced S phase cell cycle arrest by up-regulating p21 proteins expression and down-regulating Cyclin A2, CDK2, and Cdc25A protein expression. Furthermore, BTFS caused DNA damage and activated the ATM-Chk2 signaling pathway to block cell cycle progression. Moreover, BTFS trigged both extrinsic and intrinsic apoptosis—BTFS up-regulated the expression of death receptor pathway-related proteins DR5, Fas, and FADD and increased the ratio of pro-apoptotic/anti-apoptotic proteins of the Bcl-2 family. BTFS-induced apoptosis seems to be related to the AKT-MDM2-p53 signaling pathway. In summary, our results demonstrate that BTFS has the potential to be used as a nutraceutical for the prevention and treatment of ovarian cancer.

## 1. Introduction

Ovarian cancer is a malignant tumor of the female reproductive system that causes high mortality in women. Worldwide, 4.4% of cancer-related deaths are due to ovarian cancer [1]. Recent statistics have shown that there were 52,100 new cases of ovarian cancer and about 22,500 deaths in China [2]. In the US, there were 22,240 new cases of ovarian cancer with a death toll of 14,070; the survival rate is only 37% [3]. Currently, the standard treatment for ovarian cancer is radical surgery combined with adjuvant chemotherapy drugs, which has certain effects on early patients. In clinic, most ovarian cancer patients are diagnosed in the middle and late stages due to the vague early symptoms and the lack of detection methods. Accordingly, ovarian cancer is easily prone to relapse and drug resistance, and ultimately leads to high mortality [4]. Thus, it is particularly necessary to find new compounds from natural products, which could effectively prevent and treat ovarian cancer.

Infinite proliferation is the main feature of cancer cells and drugs that inhibit cell division are often used in cancer treatment. Disorders of the cell cycle can cause cells to proliferate indefinitely and induce cancer. Cyclin dependent kinases (CDKs), Cyclin, and CKI are highly correlated with the occurrence, development, and prognosis of ovarian cancer [5]. CDK2, CDK4, Cyclin A, Cyclin D, and the expression of Cyclin E are upregulated significantly in ovarian cancer tissues [6], while the protein expression of p21 and p27 are significantly lower than that of non-cancerous ovarian tissue. Many studies have improved the therapeutic effect through the action of drugs on cell cycle checkpoints. Apoptosis is a kind of programmed cell under physiological conditions. The abnormal regulation of apoptosis is closely related to the occurrence and development of cancer. Targeting extrinsic (death receptor-mediated) and intrinsic (mitochondrial-mediated) apoptosis is an important strategy in cancer treatment [7].

Saponins are a class of glycoside compounds with polycyclic compounds as ligands that are widely present in ginseng [8], Chonglou [9], Camellia [10], and other plants. Recent studies demonstrate that saponins show a strong inhibitory effect against ovarian cancer. Chonglou Saponin II induced G2 phase cell cycle arrest in SKOV3 ovarian cancer cells [11]. Baiying steroidal saponin aescin up-regulates the activity of Caspase-3 and promotes the apoptosis of SKOV3 cells [12]. Moreover, several other studies have demonstrated the potency of saponins by inhibiting cell proliferation, inducing apoptosis and cell cycle arrest, as well as inhibiting angiogenesis and weakening the invasion and metastasis, which has made saponins important natural sources in the search for new chemopreventive agents [13,14,15].

Tea is a popular beverage globally, and many functional ingredients have multiple health functions for the human body. Tea saponins are widely distributed in the roots, stems, leaves, and flowers [13]. However, compared to tea leaves, tea flowers contain a much higher amount of saponins. Moreover, tea flowers have become important food resources and it is an urgent need to explore the health benefits of tea flowers saponins. In recent years, tea flower saponins have demonstrated strong anticancer activity, as confirmed by several research. Kitagawa et al. showed that Chakasaponin I, II, and Floratheasaponin A have inhibitory effects on human gastrointestinal cancer cells HSC-2, HSC-4, MKN-45, and Caco-2, and the effect is stronger than catechin, flavonoids, and caffeine. The inhibition of HSC-2 cell proliferation may be due to the induction of cell cycle arrest in the G2/M phase and activation of apoptosis [16]. Previously, we also reported the effects of tea flower saponins containing Chakasaponin II and other 13 monomers on ovarian cancer cells; the results demonstrated that tea flower saponins shows strong anticancer activity by inducing apoptosis and S phase cell cycle arrest in human OVCAR-3 and A2780/CP70 cancer cells [17]. Now, further-purified tea flower saponins is needed to better elucidate anticancer properties and mechanisms. Thus, the aim of this paper is to obtain high-purity tea flower saponins and further elucidate its antiproliferative effect and mechanisms on human ovarian cancer cell. Our results show that high-purity BTFS extracted from Baiye No.1 tea flowers induced cell cycle arrest in the S phase and induced apoptosis in A2780/CP70 cells by targeting both extrinsic and intrinsic apoptotic pathways.

## 2. Results

### 2.1. Analysis and Identification of BTFS

**Figure 1:**
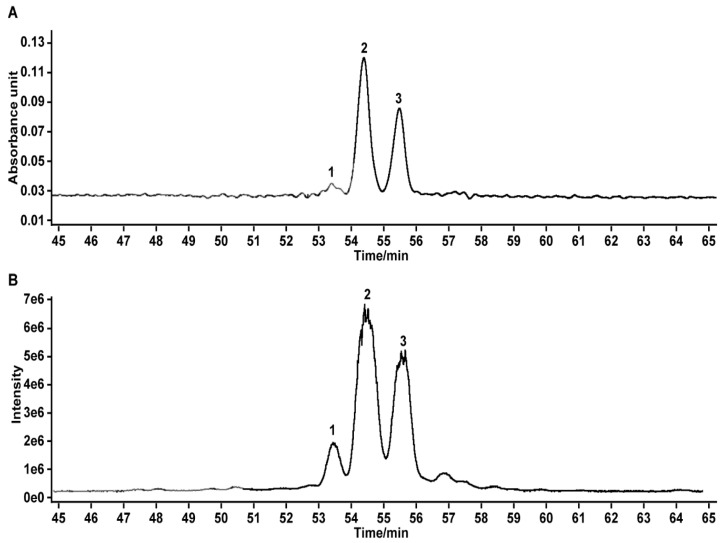
Typical chromatograms of BTFS. (**A**) Ultraviolet chromatograms. (**B**) Total ion chromatograms.

We found, through UPLC-Q-TOF/MS/MS analysis, that BTFS extracted from tea flowers contained 3 triterpenoid saponins (Figure 1A,B). Based on the published literature [18,19,20] and combined with the retention time, molecular weight, molecular formula, and secondary ion fragment information of the corresponding peak, we inferred that the saponins in BTFS are Floratheasaponin D (58.5%), Floratheasaponin A (36.7%), and an unnamed saponin (4.9%) (Table 1) [20]. Our previous study showed that Floratheasaponin A and Floratheasaponin D were the main saponin components in Baiye No.1 tea flowers [20].

### 2.2. BTFS Inhibits Ovarian Cancer Cell Proliferation

**Figure 2:**
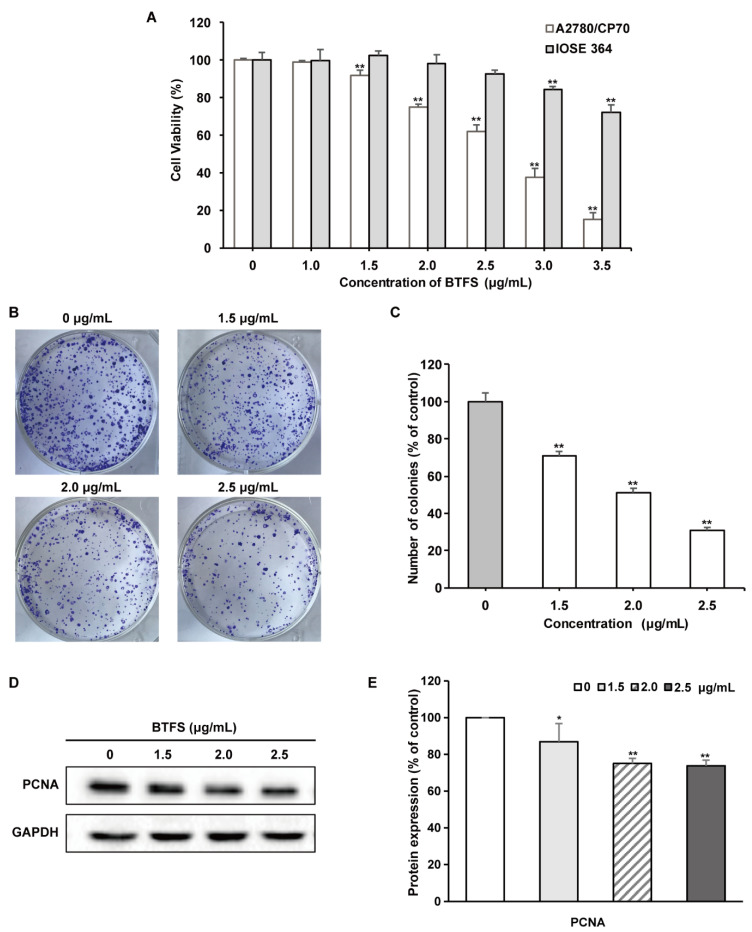
BTFS inhibits proliferation of A2780/CP70 cells. (**A**) BTFS inhibited the cell viability of A2780/CP70 and showed moderate effect on IOSE-364 cells. (**B**) The effect of BTFS on colony formation of A2780/CP70 cells. (**C**) Statistical histogram. *p < 0.05; **p < 0.01 versus control. (**D**)Effects of BTFS on protein expression of PNCA in A2780/CP70 cells. (**E**) Statistical histogram of protein quantization. * *p* < 0.05; ** *p* < 0.01 versus control.

The effects of BTFS on cell viability were determined by CellTiter 96 Aqueous One Solution Cell Proliferation assay. BTFS reduced the cell viability of A2780/CP70 cells dose-dependently, while BTFS displayed less cytotoxicity to normal ovarian cell line IOSE-364 (Figure 2A). Colony formation assay is considered as an effective way to determine the long-term proliferation capacity of cancer cells. We then carried out this assay to further explore the inhibitory effect of BTFS on A2780/CP70 cells. The results found that the number of cell colonies was significantly lower for cells subjected to BTFS than for the control cell (Figure 2B,C). Besides, we also analyzed the protein expression of proliferating cell nuclear antigen (PCNA) by Western blot; the results showed that PCNA was downregulated in the BTFS-treated group, compared to the vehicle group. (Figure 2D,E, the original images for Figure 2D can be seen in Supplementary Materials). In summary, the results demonstrated that BTFS exhibited an antiproliferative effect and cytotoxicity on A2780/CP70 cells.

### 2.3. BTFS Induces Cell Cycle Arrest in the S Phase in A2780/CP70 Cells

**Figure 3:**
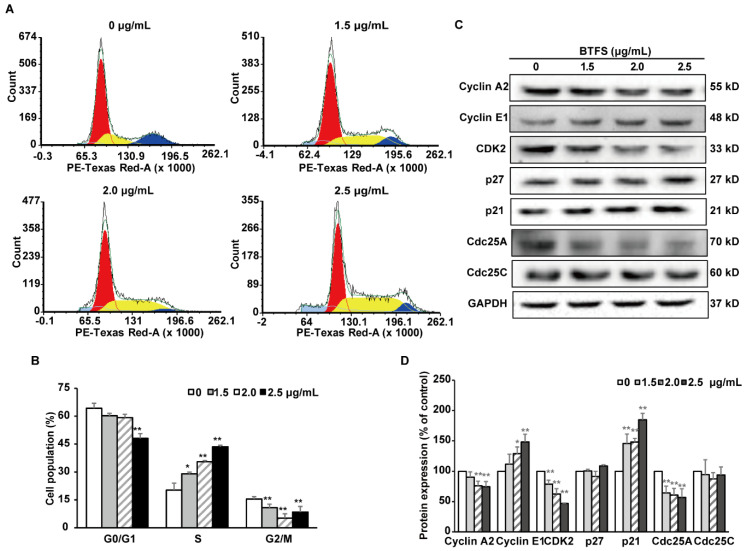
BTFS-induced cell cycle arrest at S phase in A2780/CP70 cells and regulated proteins expression related with S phase. (**A**,**B**) BTFS induced cell cycle arrest at S phase by flow cytometry. Statistical analysis bar chart, * *p* < 0.05 and ** *p* < 0.01 versus control. (**C**,**D**) Effects of BTFS on the expression of cell cycle-related proteins in A2780/CP70 cancer cells. Statistical histogram of protein quantization, * *p* < 0.05; ** *p* < 0.01 versus control.

In order to elucidate the mechanism of BTFS inhibiting cell proliferation, flow cytometry was used to detect the cell cycle phase distribution of BTFS-treated human ovarian cells stained with propidium iodide (PI). The results showed that BTFS treatment induced a dose-dependent increase in the proportion of A2780/CP70 cells in the S phase. We also observed a reduction of cell proportion in the G0/G1 and G2/M phases (Figure 3A,B). When cells were treated with 1.5, 2.0, and 2.5 μg/mL BTFS for 24 h, the proportion of cells at the S phase were 29.04%, 35.60%, and 43.52%, respectively, compared with 20.30% in the vehicle-treated cells.

### 2.4. The Effects of BTFS on Cell Cycle Regulatory Protein Expression

We then evaluated the expression of cell cycle regulatory proteins by western blot after BTFS treatment. Cyclin and Cyclin dependent kinase (CDK) form Cyclin/CDK complex to regulate cell cycle progression. Furthermore, p21 and p27 are CDK inhibitors (CDKI), which negatively regulate cell cycle; the Cdc25 phosphatase family have a positive regulation effect on cell cycle. We found that BTFS could effectively suppress the expression of CDK2, Cyclin A, and Cdc25A and increased the expression of Cyclin E1 and p21, while showing no effect on the protein levels of p27 and Cdc25C (Figure 3C,D). The results demonstrated that the down-regulation of Cdc25A, up-regulation of p21, and reduction of kinase activities of CyclinE1/CDK2 and CyclinA2/CDK2 complexes might be responsible for S phase arrest induced by BTFS in A2780/CP70 cells.

### 2.5. BTFS Activates Apoptosis in A2780/CP70 Cells

**Figure 4:**
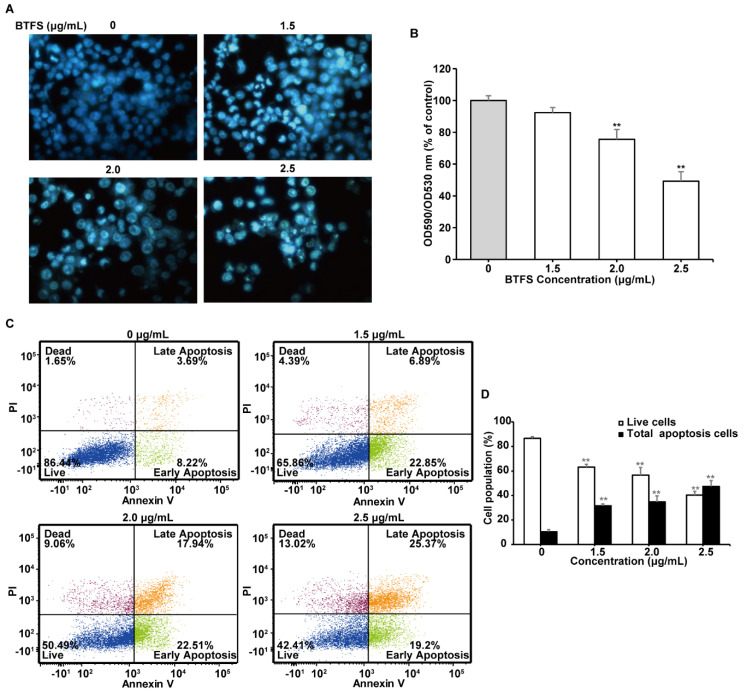
BTFS-induced apoptosis in A2780/CP70 cells. (**A**) Hoechst 33342 staining confirmed the apoptotic effect induced by BTFS in A2780/CP70 cells. (**B**) The effect of BTFS on mitochondrial membrane potential in A2780/CP70 cells was determined by JC-1 staining. ** *p* < 0.01 versus control. (**C**,**D**) BTFS-induced apoptosis in A2780/CP70 cells evidenced by flow cytometry. Statistical analysis bar chart, ** *p* < 0.01 versus control.

The increasing of sub-G1 phase population demonstrates that BTFS might cause cellular apoptosis in A2780/CP70 cells. Accordingly, we further explored whether BTFS caused apoptosis in A2780/CP70 cells. Hoechst 33342 staining was performed to observe the morphological changes of apoptosis. As shown in Figure 4A, after treating with BTFS, A2780/CP70 cells showed more apoptotic cells, which were brighter blue with condensed or fragmented nuclei than the untreated group. We then used quantitative fluorescence spectrophotometer to evaluate the mitochondrial membrane potential of A2780/CP70 cells. BTFS treatment resulted in a notable reduction in the red-green fluorescence ratio of JC-1 dye, which indicated that BTFS could induce apoptosis of A2780/CP70 cells (Figure 4B). Flow cytometric analysis was then conducted to further verify the pro-apoptotic effect of BTFS. As shown in Figure 4C,D, BTFS could significantly reduce the proportion of live cells and increase the proportion of apoptotic cells dose-dependently. Together, these results suggested that inducing apoptosis may be an important factor for the antiproliferative effect of BTFS on A2780/CP70 cells.

### 2.6. BTFS Mediates Apoptosis via Intrinsic and Extrinsic Apoptotic Pathways

**Figure 5:**
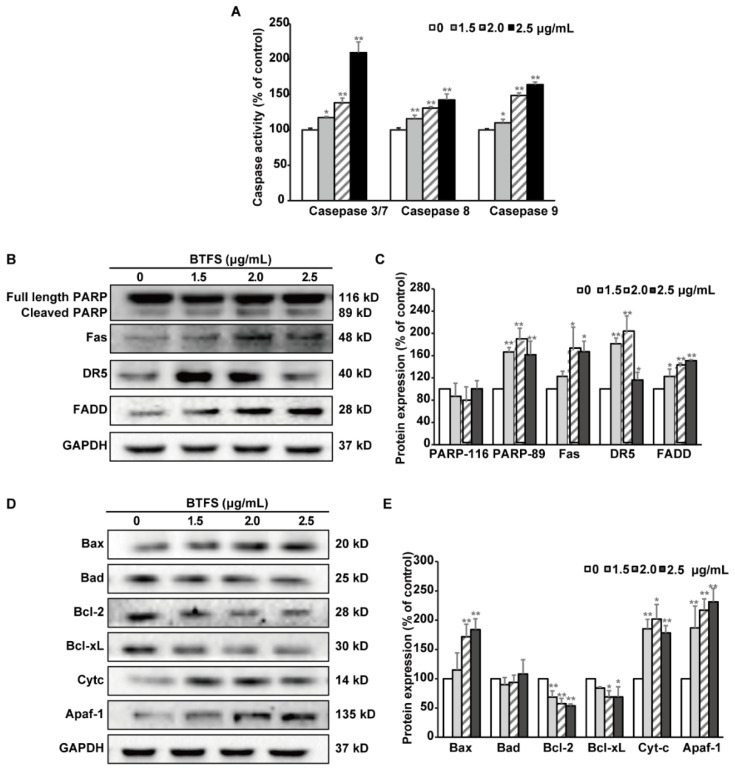
BTFS trigged both extrinsic and intrinsic pathways. (**A**) Effects of BTFS on the activity of caspase-3/7, 8 and 9. * *p* < 0.05 and ** *p* < 0.01 versus control. (**B**,**C**) Effects of BTFS on extrinsic apoptosis-related protein expression in A2780/CP70 cells. (**D**,**E**) Effect of BTFS on intrinsic apoptosis-related protein expression in A2780/CP70 cells.

Apoptosis could be classified into the intrinsic (mitochondrial mediated) pathway and the extrinsic (death receptor mediated) pathway. To investigate the specific pathway of BTFS in inducing apoptosis in A2780/CP70 cells, caspase-Glo assay was used to detected the caspase activation. As shown in Figure 5A, caspase-3/7, -8 and -9 activities in BTFS-treated cells were increased significantly compared with vehicle treated cells, which hinted that BTFS might trigger both the intrinsic and extrinsic apoptotic pathway. Furthermore, western blot assay was then conducted to examine the key proteins in both apoptotic pathways. After BTFS treatment, cleaved PARP protein, served as a marker of apoptosis, increased significantly, further confirming that BTFS could induce apoptosis in A2780/CP70 cells. Meanwhile, extrinsic pathway related proteins were investigated to confirm whether the extrinsic pathway was involved in BTFS-induced apoptosis. The results showed that BTFS could markedly up-regulate the expression of death receptors, Fas and DR5, and dose-dependently increased the cell death adaptor protein FADD (Figure 5B,C). With regard to the intrinsic apoptosis-related proteins, BTFS obviously decreased the protein expression of Bcl-2 and Bcl-xL, while Bax, Cytochrome C, and Apaf apoptotic protease activating factor-1(Apaf-1) were significantly upregulated and had no effect on Bad expression (Figure 5D,E). These results suggested that both intrinsic and extrinsic pathways were involved in BTFS-induced apoptosis in A2780/CP70 cells.

### 2.7. BTFS Induces DNA Damage and Affects the Expression of Upstream Regulators AKT, MDM2, and P53

**Figure 6:**
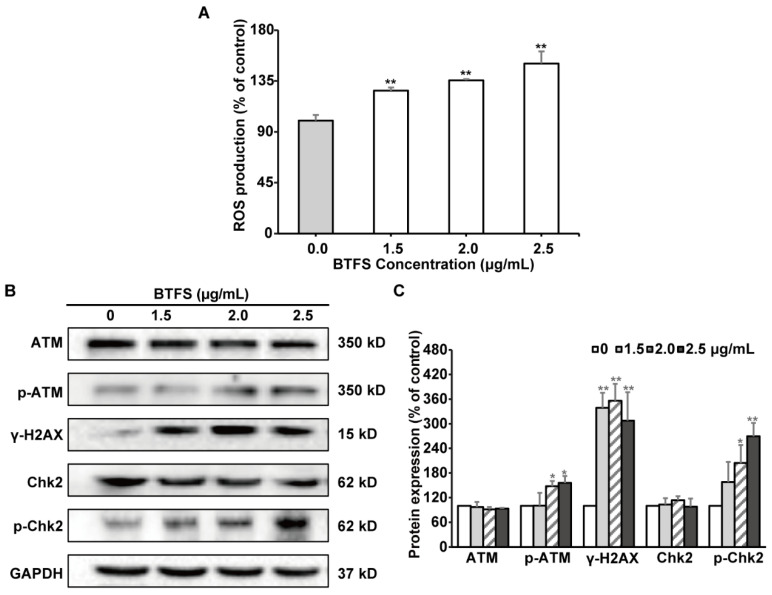
BTFS caused DNA damage in A2780/CP70 cells. (**A**) The effect of BTFS 3 on ROS production in A2780/CP70 cells. * *p* < 0.05; ** *p* < 0.01 versus control. (**B**,**C**) The effect of BTFS on the expression of DNA damage-related proteins in A2780/CP70 cells. * *p* < 0.05; ** *p* < 0.01 versus control.

ROS and DNA damages could mediate cell cycle arrest and apoptosis [21,22]. Firstly, DCFH-DA fluorescence probe assay was conducted to assess the effect of BTFS on intracellular ROS production. ROS generation was obviously elevated when A2780/CP70 cells were exposed to BTFS, compared to the vehicle-treated group (Figure 6A). Next, western blot assay was then performed to investigate the effect of BTFS on DNA damage response. The phosphorylation of histone H2A.X at serine 139 is considered as a reliable marker of DNA damage [23]. BTFS dramatically increased the phosphorylation of histone H2A.X. ATM/Chk is one of the important signaling pathways mediating DNA damage. After exposure to BTFS, both the phosphorylation of ATM at Ser1981 as well as the phosphorylation of Chk2 at Thr68 were up-regulated (Figure 6B,C), suggesting that ATM-Chk2 pathway was activated. These results suggested that BTFS promoted ROS generation and DNA damage, which is responsible for the cytotoxicity of BTFS. Moreover, BTFS induced DNA damage may be mediated by ROS generation, the specific mechanism of which needs to be further elucidated.

**Figure 7:**
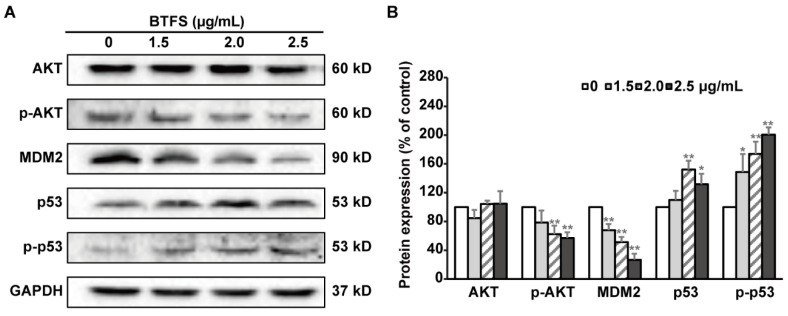
Effect of BTFS on AKT/MDM/P53 pathway related proteins. (**A**) Western blot image (**B**) Statistical histogram of protein quantization. * *p* < 0.05; ** *p* < 0.01 versus control.

AKT, MDM2, and p53 are upstream regulators of the cell cycle and apoptosis related proteins mentioned above. To further explore the mechanism underlying BTFS-induced S phase cell cycle arrest and apoptosis, we evaluated the protein expression levels of these upstream regulators in A2780/CP70 cells after BTFS exposure. Western blot analysis illustrated that p-AKT and MDM2 were dramatically decreased by BTFS, meanwhile p53 and p-p53 were increased compared with the controls (Figure 7A,B).

## 3. Discussion

Ovarian cancer is considered to be one of the deadliest gynecological cancers in the world. Although platinum-based chemotherapy is the most broadly used treatment for this disease, chemoresistance and adverse side-effects still remain major obstacles to successful treatment [24]. Compounds derived from natural products have recently gained much attention in cancer therapy due to the higher biological activity and lower toxicity [25]. It has been proved that various triterpenoid saponins have extensive anticancer activity [14,15] and some triterpenoid saponins, such as Albizia gummifera saponins [26], ginsenoside 20(S)-Rg3 [27], ginsenoside Rh2 [28], Camellia oleifera Abel seed saponins [29], and *Camellia sinensis* seed saponins [30], exhibited obvious anti-proliferative effect against ovarian cancer cells. In this study, we extracted and isolated the characteristic saponin complex BTFS from Baiye No.1 tea flowers, which mainly contained Floratheasaponin A and Floratheasaponin D, and found that BTFS strongly inhibited cell proliferation of human ovarian cancer cells A2780/CP70 by induction of S phase cell cycle arrest and apoptosis.

This research measured the cytotoxic effect of BTFS on ovarian cells and found that BTFS significantly reduced the viability of A2780/CP70 cells, whereas it hardly affected the normal human immortalized ovarian surface epithelial IOSE 364 cells at indicated concentrations. Ovarian cancer cells have unlimited proliferation ability, while healthy cells normally proliferate. BTFS can inhibit cell proliferation by inducing apoptosis and S-phase cycle arrest in A2780/CP70. BTFS may not cause apoptosis and cycle arrest of normal cells. This may be why BTFS is selective towards ovarian cancer cells. The IC50 value of BTFS on A2780/CP70 cells was calculated to be 2.6 μg/mL, lower than theaflavin-3,3′-digallate (IC50 was about 20.7 μg/mL on A2780/CP70 cells) [31], and tea seed saponins (IC50 was about 5.9 μg/mL on OVCAR-3 and A2780/CP70 cells) [30]. Besides, the results obtained from colony formation assay and the Western blot of PCNA further demonstrated that BTFS had obvious inhibitory effects on A2780/CP70 cells.

The cell cycle disorder leading to the abnormal cell proliferation is one of the main mechanisms of tumorigenesis [32]. The S phase in the DNA synthesis phase is when DNA is replicated; thus, it is a pivotal part of the cell cycle [33]. The cell cycle is a sequential process and any phase of arrest will cause cell proliferation stagnation or death. Numerous chemotherapeutic agents exert anticancer properties by interfering with the cell cycle. In our research, analysis of the distribution of cell cycle showed that BTFS could cause S phase cell cycle arrest in A2780/CP70 cells. This result was consistent with studies that many saponins extracted from natural plants also inhibit cancer cell proliferation by arresting cells in S phase, such as Astragalus saponins [34], Albiziae Cortex total saponins [35], and ginsenoside Re [36]. Cell cycle progression is regulated by cyclins, CDKs, and other regulatory proteins [37]. Cyclin E/CDK2 complex actively participates in G1/S transition and plays important role in the initial stage of S phase. S phase progression is impacted by Cyclin A/CDK2 complex, and the S/G2 transition also requires Cyclin A/CDK2 complex [38,39]. Moreover, Cyclin/CDK complexes are modulated by Cyclin-dependent kinase inhibitors and the Cdc25 phosphatase family. The p21 protein is considered as a Cyclin dependent kinase inhibitor, which inhibits the formation of Cyclins/CDK2 complex, ultimately blocking cell cycle progression from S to G2/M phase [40,41]. CDC25A, a dual specificity phosphatase, activates the Cyclin/CDK complexes, which promotes cell cycle progression, and overexpression of CDC25A will lead to abnormal cell cycle regulation and lead to tumorigenesis [42,43]. In this study, after BTFS treatment, Cyclin A2, CDK2, and Cdc25A were downregulated significantly, while p21 and Cyclin E1 proteins were significantly upregulated. Thus, these data suggest that BTFS-induced S phase arrest might be due to changes in the S phase related proteins in A2780/CP70 cells.

Apoptosis is considered one of the most widespread forms of programmed cell death, playing a pivotal role in various physiological processes and pathological conditions [44]. Inducing apoptosis is regarded as one of the major mechanisms for cancer treatments with natural compounds. In this study, the results obtained from Hoechst 33342 staining, JC-1 fluorescent staining, and flow cytometry assay suggest that BTFS induced apoptosis in A2780/CP70 cells as evidenced by much brighter and more condensed nuclei within cells, notable reduction of mitochondrial membrane potential, and higher percentage of apoptotic cells. Two well-known pathways to trigger apoptosis are the extrinsic and intrinsic apoptotic pathways. Activation of caspase-8 and caspase-9 are regarded as essential markers of the extrinsic and intrinsic apoptotic pathways, respectively, and can further activate downstream effector caspase-3 and caspase-7, which are able to mediate the cleavage of PARP, and then regulate apoptosis [45,46]. In our research, BTFS obviously activated caspase-3/7, -8 and -9 and increased the level of cleaved PARP-1, indicating that BTFS triggered both the extrinsic and intrinsic apoptotic pathways in A2780/CP70 cells. For the extrinsic pathway, tumor necrosis factor-related apoptosis-inducing ligands (TRAIL), for instance, Apo2L/TRAIL and Fas ligand (FasL), bind with their respective death receptors, such as DR4/DR5 or Fas, and subsequently interact with the adaptor molecule, Fas-associated death domain (FADD) [47]. FADD combines with caspase-8, followed by initiating the caspase-3/7 cascade reaction and ultimately cell death [48]. The current research found that BTFS evidently up-regulated DR5, Fas, and FADD, suggesting activation of the extrinsic apoptotic pathway. Previous research reported that tea seeds saponins could also increase protein expression of DR5 and FADD and induced apoptosis in ovarian cancer cell, which are consistent with our results [30]. In the intrinsic pathway, the Bcl-2 family proteins, which include pro-apoptotic (Bax, Bad, and Bak) and anti-apoptotic (Bcl-2, Bcl-xL and Bcl-B) members, are key regulators by governing the mitochondrial outer membrane permeabilization (MOMP) [30]. After the decrease of mitochondrial membrane potential caused through the increase of MOMP, cytochrome c is released from the mitochondria into the cytosol, and then binds with Apaf-1 to form the apoptosome complex, which will activate pro-caspase 9 and trigger an enzymatic cascade leading to cell death [49,50]. In this case, BTFS increased the ratio of pro- versus anti-apoptotic Bcl-2 family protein and the release of cytochrome C and Apaf-1. A similar study reported that Paris saponin II induced apoptosis in ovarian cancer cell might also result from its effects on the expression of Bax, Bcl-2, and cytochrome c proteins [11]. These results demonstrate that induction of apoptosis through both extrinsic and intrinsic pathways might also account for the anti-proliferation activity of BTFS on A2780/CP70 cells. Previously we reported the anti-cancer effect of saponin extract, which contained 14 triterpenoid saponins [17]. In this paper, a higher-purity BTFS, which mainly contained Floratheasaponin D and Floratheasaponin A, was obtained and it showed strong antiproliferative effect against A2780/CP70 cells. Though BTFS could also induce apoptosis and cell cycle arrest, the specific contribution of Floratheasaponin D and Floratheasaponin A remains to be further elucidated. Moreover, structure-activity relationship may play a pivotal role in its anticancer effect. It has been reported that the differences of saponin type, position, and sugar moieties affect anticancer activity [51,52]. BTFS was classified as triterpenes saponins in our study; we obtained a mixture of three saponin monomers, which makes it complex to evaluate the anticancer effect and the chemical structures. However, the specific structure-activity relationship of tea flower saponins is worth investigation in future work after we obtain the monomers.

Inducing intracellular ROS generation is considered as an important mechanism of the anticancer effect of most drugs [53]. Excessive ROS generation could cause DNA double-strand break, DNA locus mutation, and other forms of DNA damage [54]; and elevated ROS levels have been regarded as a causative trigger for DNA damage [55]. Cell cycle checkpoints and effector kinases could be activated to regulate cellular decision among cell cycle arrest, apoptosis, or other cell death modalities in response to ROS-mediated DNA damage [56]. In our research, we demonstrated ROS generation and DNA damage after treatment with BTFS in A2780/CP70 cells. Ataxia telangiectasia mutated (ATM) kinase is an important DNA damage sensor for oxidative stress response. When responding to DNA damage, ATM is activated by autophosphorylation and further phosphorylates downstream substrates such as Chk1 and Chk2 [57,58]. Furthermore, activated Chk1 and Chk2 cause the phosphorylation of their downstream effectors, for instance Cdc25A, which could mediate the transformation of cell cycle S/G2 by regulating CDK2 activity [59]. Moreover, Chk2 could phosphorylate and activate p53 protein directly [60]. This experiment revealed that p-ATM and p-Chk2 were remarkably increased by BTFS in A2780/CP70 cells, suggesting that the ATM-Chk2 pathway was activated following DNA damage.

A serine/threonine kinase named AKT can be activated by autophosphorylation at Ser473 and Thr308, thereby inhibiting apoptosis and promoting cell survival. MDM2 is one of the downstream proteins of AKT, which can promote cell proliferation and growth, and is related to the development of a variety of tumors [61]. MDM2 is also a ubiquitin ligase of p53, which can be phosphorylated by p-AKT at Ser166 and Ser186 and enter the nucleus to form a complex with p53, thereby inhibiting p53 activity [62]. P53 is an important tumor suppressor, which plays an important role in cellular response. DNA damage can induce p53 phosphorylation at Ser15 and Ser20 sites, weakening the interaction between p53 and MDM2 and inducing apoptosis, cell cycle arrest, etc. [62,63]. P53 mainly induces apoptosis by activating the death receptor pathway and the mitochondrial pathway; meanwhile, p53 can promote apoptosis by activating death receptors such as Fas, DR4, and DR5 located on the cell membrane. On the other hand, Bcl-2 family proteins as well as mitochondria proteins are also associated with p53-dependent apoptosis [64]. It has been reported that triterpenoid saponin ginsenoside Rh2 can activate the p53 pathway to induce apoptosis in colorectal cancer cells [64], and can induce apoptosis in human epidermal cancer cells by inhibiting AKT activity [65]. Based on our study, we found that BTFS can significantly reduce p-AKT and MDM2 and increase p53 and p-p53 protein levels in A2780/CP70 cells, suggesting that BTFS can reduce the cross-linking of MDM2 and p53 by inhibiting AKT autophosphorylation, and thus effectively enhance p53 activity and promote cell apoptosis. Our finding is consistent with the study that the active tea component theaflavins can induce apoptosis of ovarian cancer cellA2780/CP70 through AKT-MDM2-p53 pathway [31].

## 4. Materials and Methods

### 4.1. Materials and Reagents

RPMI-1640 medium, Fetal bovine serum (FBS), and DMSO were purchased from Sigma (St. Louis, MO, USA). Caspase-Glo 3/7, Caspase-8, Caspase-9, and CellTiter 96 Aqueous kit were purchased from Promega (Madison, WI, USA). Propidium iodide (PI) and Alexa Fluor 488 Annexin V were purchased from Invitrogen (Waltham, MA, USA). Antibodies against PCNA, p21, p27, CDK2, Cdc25A, Cdc25C, CyclinA2, Cyclin E1, ATM, p-ATM (Ser1981), γ-H2AX (Ser139), PARP, Fas, DR5, FADD, Bax, Bcl-2, Bcl-xL, Apaf-1, Cytc, p53, p-p53 (Ser15), AKT, p-AKT (Ser473), and MDM2 were purchased from Cell Signaling Technology (Beverly, MA, USA). Antibodies against Chk2, p-Chk2 (Thr 68), Bad, and GAPDH were purchased from Santa Cruz Biotechnology (Dallas, TX, USA).

### 4.2. Extraction and Identification of Baiye No.1 Tea Flower Saponins (BTFS)

Dried tea flowers of tea Baiye No.1 variety were obtained from Zhe-jiang Yilongfang Co., Ltd. (Kaihua, Zhe-jiang, China). After smashing, the powder was distilled at 80 °C for 45 min. The aqueous extract was separated by AB-8 macroporous adsorption resin, followed by preparative HPLC to obtain BTFS. BTFS was then identified by UPLC-Q-TOF/MS/MS system with ultra-high performance chromatography (Agilent, Santa Clara, CA, USA) coupled with electro-spray ionization quadrupole time-of-flight mass spectrometry (AB SCIEX, Framingham, MA, USA) according to the methods we described previously, with certain modifications [20]. BTFS was dissolved in dimethyl sulfoxide (DMSO) to make a 20 mg/mL stock solution and stored at −20 °C before use.

### 4.3. Cell Lines and Cell Culture

Human ovarian cancer cell line A2780/CP70 was provided by Dr. Bing-Hua Jiang from Thomas Jefferson University, and the normal ovarian surface epithelial cell line IOSE-364 was provided by Dr Auersperg from the University of British Columbia. The cell lines were cultured in RPMI-1640 medium with 10% FBS, and incubated at 37 °C in a humidified incubator with 5% CO_2._

### 4.4. Cell Viability Assay

A2780/CP70 and IOSE-364 cells were seeded in 96-well plates at a density of 2 × 10^4^ cells/well incubated overnight, and treated with BTFS (1.0, 1.5, 2.0, 2.5, 3.0, 3.5 μg/mL) or an equal concentration of DMSO (as vehicle) for 24 h. CellTiter 96 Aqueous kit was then used to assess the cell viability according to the manufacturer’s protocol. Results were presented as percentage of control.

### 4.5. Colony Formation Assay

A2780/CP70 cells were seeded into 6-well plates at 6 × 10^5^ cells/well incubated overnight and treated with BTFS (0, 1.5, 2.0, 2.5 μg/mL) for 24 h. The cells were then cultured for 7 days with drug-free medium at 2 × 10^3^ cells/well. Colonies were fixed with ice-cold methanol for 10 min, then stained with 0.5% crystal violet solution in 25% methanol for another 10 min. The plates were rinsed with distilled water carefully several times and directly photographed. Image J software (Bethesda, MD, USA) was used to count the number of colonies; the results were adjusted to the percentage of control.

### 4.6. Cell Cycle Analysis by Flow Cytometry

After treating with BTFS (0, 1.5, 2.0, 2.5 μg/mL) for 24 h, the cells were washed with cold PBS twice and then fixed with ice-cold 70% ethanol at 4 °C overnight. Afterwards, the cells were washed twice with PBS and incubated with 180 μg/mL RNase (Invitrogen) for 15 min at 37 °C, then incubating with 50 μg/mL propidium iodide (PI) solution (Sigma) for 15 min in the dark at 37 °C. The cells were then analyzed by FACSCalibur flow cytometry (BD Biosciences, San Jose, CA, USA) and data were analyzed by FCS software (De Novo Software, CA, USA).

### 4.7. Hoechse 33342 Staining

The cells were seeded in 96 well plates at density of 2 × 10^4^ cells/well and incubated overnight, after treating with BTFS (0, 1.5, 2.0, 2.5 μg/mL) for 24 h. The cells were stained with 10 µg/mL Hoechst 33342 (Sigma) in PBS for 15 min in the dark at 37 °C. Fluorescence microscope (ZEISS, Heidelberg, Germany) was used to examine the cellular morphology.

### 4.8. Evaluation of Mitochondrial Membrane Potential

The cells were seeded in black 96 well plates at density of 2 × 10^4^ cells/well and incubated overnight, and treated with BTFS (0, 1.5, 2.0, 2.5 μg/mL) for 24 h. After washing twice, the cells were incubated with 10 µg/mL JC-1 solution for 30 min at 37 °C. The fluorescent intensity was measured using Synerg HT Multi Mode Microplate reader (BioTek, Winooski, VT, USA) at excitation: emission of 485/590 and 485/528 for red aggregates and green monomers, respectively.

### 4.9. Apoptosis Analysis by Flow Cytometry

The apoptotic cells were evaluated using an Alexa Fluor 488 Annexin V/Dead Cell Apoptosis Kit (Invitrogen, Waltham, MA, USA). After treating with BTFS (0, 1.5, 2.0, 2.5 μg/mL) for 24 h, the cells were harvested and centrifuged for 8 min at 1000 rpm; after washing twice with PBS, the cells were suspended in Annexin-binding buffer with Alexa Fluor 488 Annexin V and PI solution for in dark 15 min at room temperature. The stained cells were then analyzed by FACSCalibur flow cytometry (BD Biosciences) with fluorescence emission at 530 nm and excitation at 488 nm.

### 4.10. Cellular Caspase Activity Assay

The cells were seeded in 96 well plates at density of 2 × 10^4^ cells/well and incubated overnight, after treating with BTFS (0, 1.5, 2.0, 2.5 μg/mL) for 24 h. Caspase Glo-3/7, -8 and -9 regents (Promega, Madison, WI, USA) were added to each well and incubated for 30 min at 37 °C. Luminescence was measured by Synerg HT Multi Mode Microplate reader (BioTek). Total protein levels were detected with a BCA assay kit to normalize the caspases activities. The results were presented as percentage of control.

### 4.11. Detection of Intracellular ROS Production

Intracellular ROS production was measured by peroxide-sensitive fluorescent probe DCFH-DA. The cells were treated with BTFS (0, 1.5, 2.0, 2.5 μg/mL) for 24 h, followed by treatment with 10 μM DCFH-DA for 30 min at 37 °C. The fluorescence intensity was measured with excitation at 485 nm and emission at 528 nm by Synergy HT Multi-Mode Microplate Reader (BioTek). Total protein levels were detected by BCA assay kit to normalize the ROS generation. The results were presented as percentage of control.

### 4.12. Western Blotting

Cells were treated with BTFS (0, 1.5, 2.0, 2.5 μg/mL) for 24 h and then harvested and extracted with Mammalian Protein Extraction Reagent (Pierce, Rockford, IL, USA) supplemented with Halt Protease and Phosphatase Inhibitor (Pierce). BCA protein assay kit (Pierce) was used to determine the total protein concentrations of cell lysates. Protein samples of cell lysates were mixed with equal amount of SDS loading buffer and denatured by heating for 8 min, then separated by SDS-polyacrylamide gel electrophoresis before transferring onto nitrocellulose membranes. The membranes were blocked with 5% non-fat milk in Tris-buffer saline, which contained 0.1% Tween 20 (TBST) for 1 h at room temperature. Specific primary antibodies were used and the membrane was incubated at 4 °C overnight. After washing with TBST, appropriate secondary antibodies conjugated with horseradish peroxidase were used. The antigen-antibody complex was visualized with the ECL Western blot detection kit (Bio-Rad) and ChemiDoc MP System (Bio Rad, Hercules, CA, USA). Image J software was used to quantitate the protein bands; the indicated protein was then normalized by GAPDH.

### 4.13. Statistical Analysis

Data were presented as mean ± standard deviation (SD) for at least three independent experiments. One-way ANOVA followed with Dunnett′s test was performed by SPSS software (IBM, Version 22.0, Armonk, NY, USA). The criterion for statistical significance were *p* < 0.05 and *p* < 0.01.

## 5. Conclusions

The high-purity standardized saponin extract we obtained from Baiye No.1 tea flower, namely BTFS, shows a strong antiproliferative effect against A2780/CP70 cells at low concentrations, while causing less cytotoxicity to normal cells. BTFS caused cellular DNA damage in A2780/CP 70 cells and induced cell cycle arrest in the S phase via regulating ATM-Chk2 signaling pathway related proteins. BTFS also trigged both intrinsic and extrinsic apoptosis in A2780/CP70 cells through the AKT-MDM2-p53 signaling pathway. Based on our results, BTFS has the potential to serve as a nutraceutical for the prevention and treatment of ovarian cancer. The specific contribution of Floratheasaponins A and D to the antiproliferative activity of A2780/CP70 cells needs further investigation.

## Figures and Tables

**Table 1:** MS data in negative mode of saponins extracted from tea flower.

Peak	Retention Time (min)	[M − H]^−^	MS2	Formula	Peak Identity
1	53.40	1245.59	1083, 1065, 951, 915, 753, 709, 611	C60H94O27	Unknown [20]
2	54.45	1229.59	1083, 1067, 1049, 789, 611	C60H94O26	Floratheasaponin D [19,20]
3	55.57	1215.58	1083, 1035, 951, 933, 789, 611	C59H92O26	Floratheasaponin A [18,20]

## Supplementary Materials

The Supplementary Materials are available online.
