# Supplementary material for: Standardized Saponin Extract from Baiye No.1 Tea (Camellia sinensis) Flowers Induced S Phase Cell Cycle Arrest and Apoptosis via AKT-MDM2-p53 Signaling Pathway in Ovarian Cancer Cells
Source: Molecules. 2020 Jul 31;25(15):3515. doi: 10.3390/molecules25153515 (PMC7435957; doi:10.3390/molecules25153515)

molecules

Article

Standardized Saponin Extract from Baiye No.1 Tea (*Camellia sinensis*) Flowers Induced S Phase Cell Cycle Arrest and Apoptosis via AKT-MDM2-p53 Signaling Pathway in Ovarian Cancer Cells

Youying Tu [1], Lianfu Chen [1,2], Ning Ren [1,2], Bo Li [1], Yuanyuan Wu [1], Gary O. Rankin [3], Yon Rojanasakul [4], Yaomin Wang [5,*] and Yi Charlie Chen [2,*]

[1] Department of Tea Science, Zhejiang University, Hangzhou 310058, Zhejiang, China; youytu@zju.edu.cn (Y.T.); c.lianfu@foxmail.com (L.C.); ningren@zju.edu.cn (N.R.); drlib@zju.edu.cn (B.L.); yywu@zju.edu.cn (Y.W.)

[2] College of Health, Science, Technology and Mathematics, Alderson Broaddus University, Philippi, WV 26416, USA

[3] Department of Biomedical Sciences, Joan C. Edwards School of Medicine, Marshall University, Huntington, WV 25755, USA; rankin@marshall.edu

[4] Department of Pharmaceutical Sciences and WVU Cancer Institute, West Virginia University, Morgantown, WV 26506, USA; yrojan@hsc.wvu.edu

[5] Key Laboratory of Horticulture Plant Biology, Ministry of Education, College of Horticulture & Forestry Sciences, Huazhong Agricultural University, Wuhan 430070, Hubei, China

* Correspondence: wangym@mail.hzau.edu.cn (Y.W.); chenyc@ab.edu (Y.C.C.)

Academic Editors: Marialuigia Fantacuzzi and Marialuigia Fantacuzzi

Received: 22 June 2020; Accepted: 29 July 2020; Published: date

Original images for Figure 2 — D

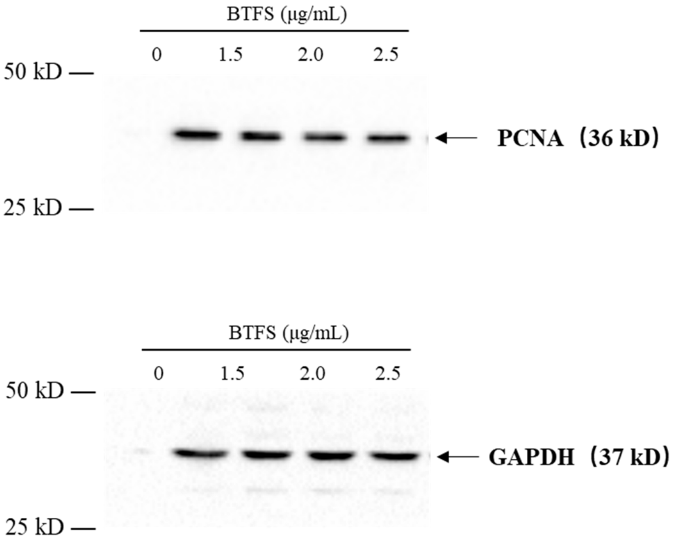

Original images for Figure 3 — C

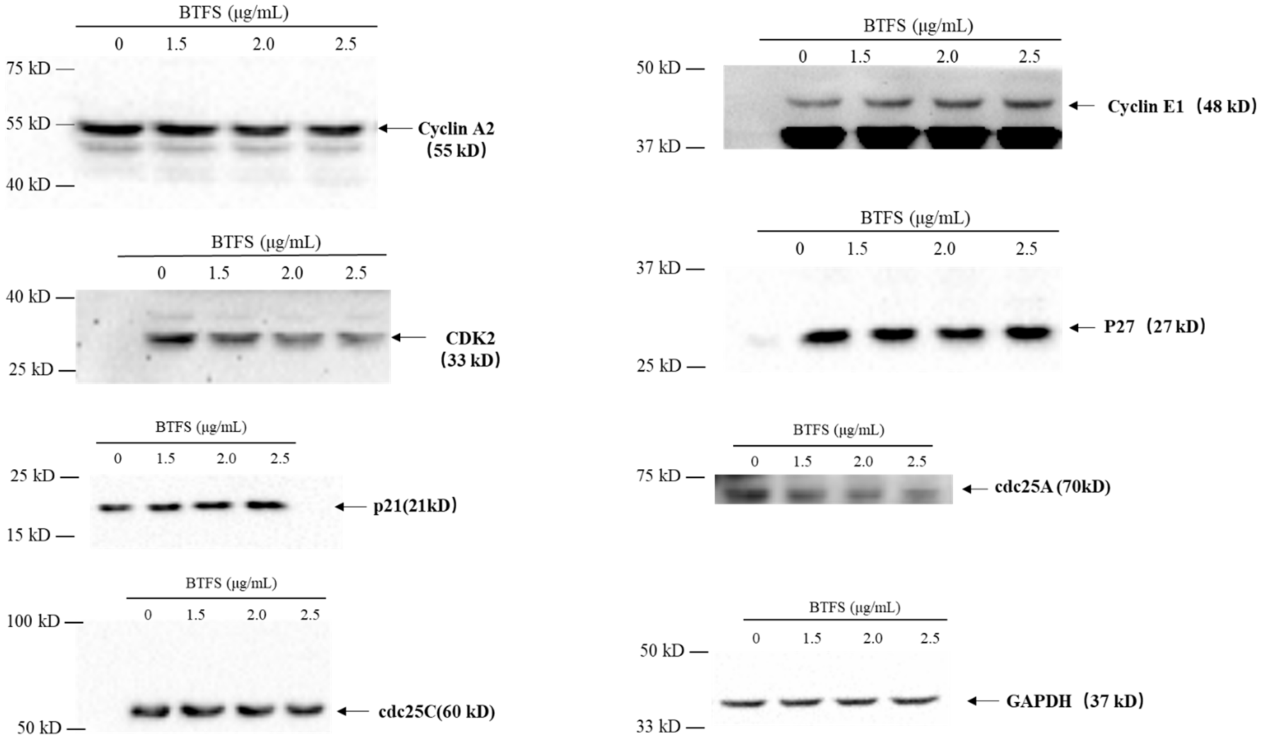

Original images for Figure 5 — B

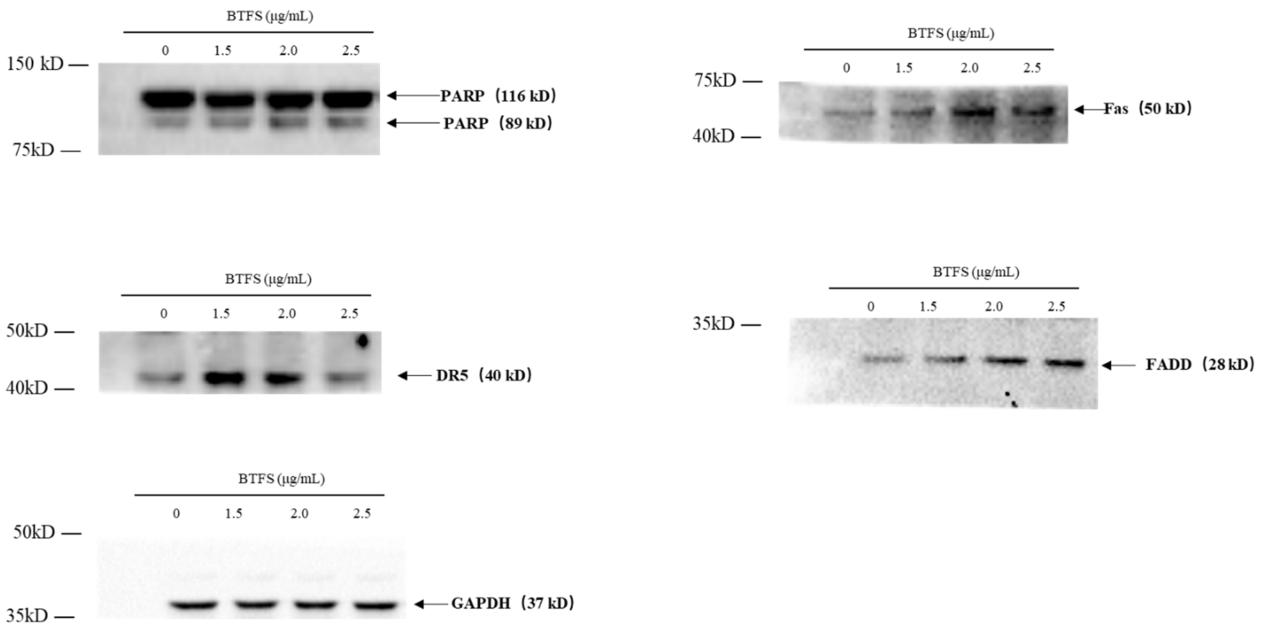

Original images for Figure 5 — D

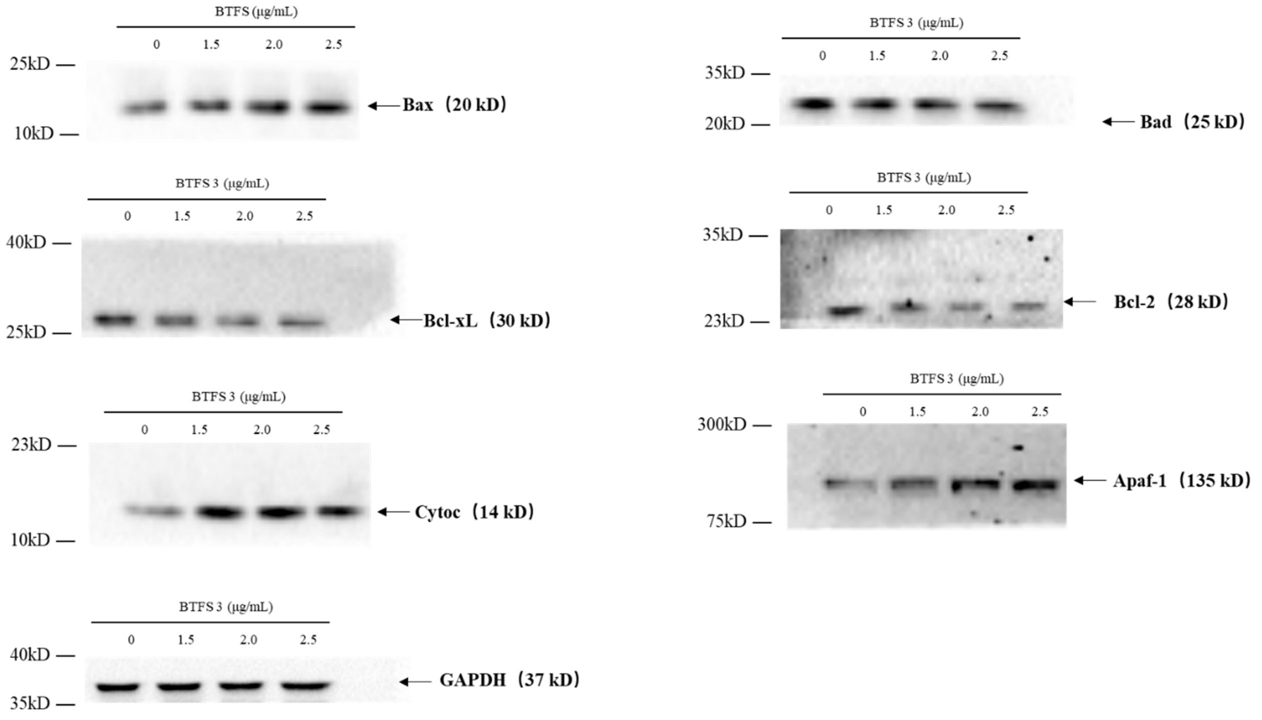

Original images for Figure 6 — B

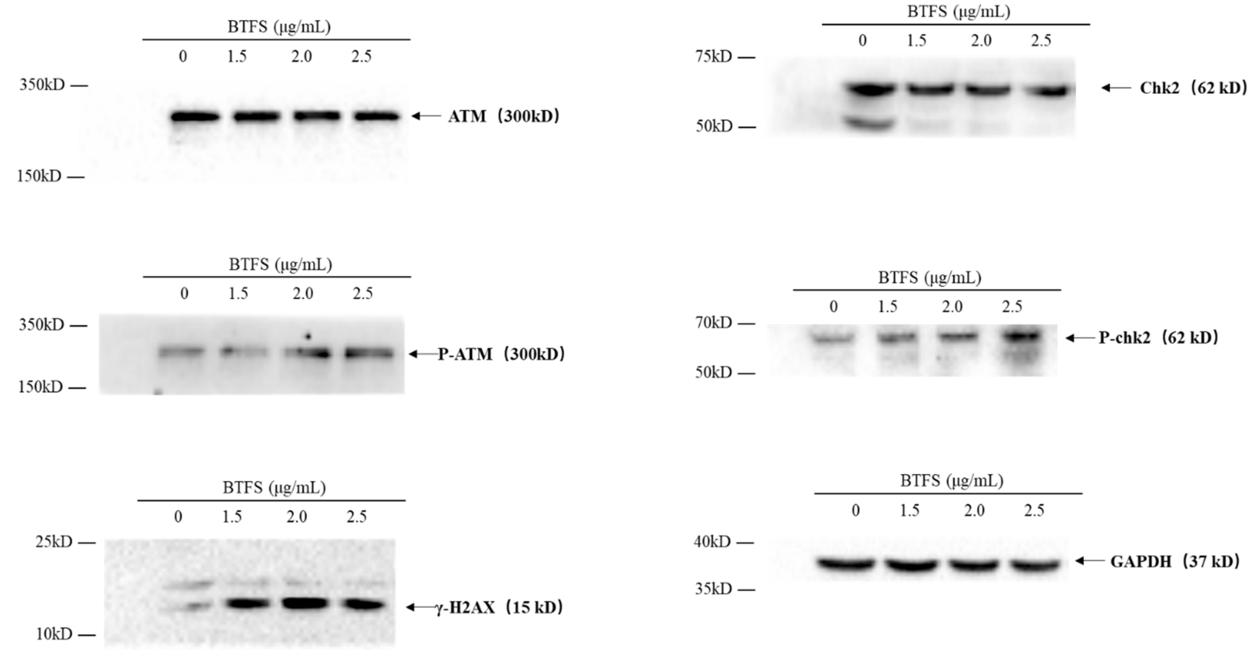

Original images for Figure 7 — A

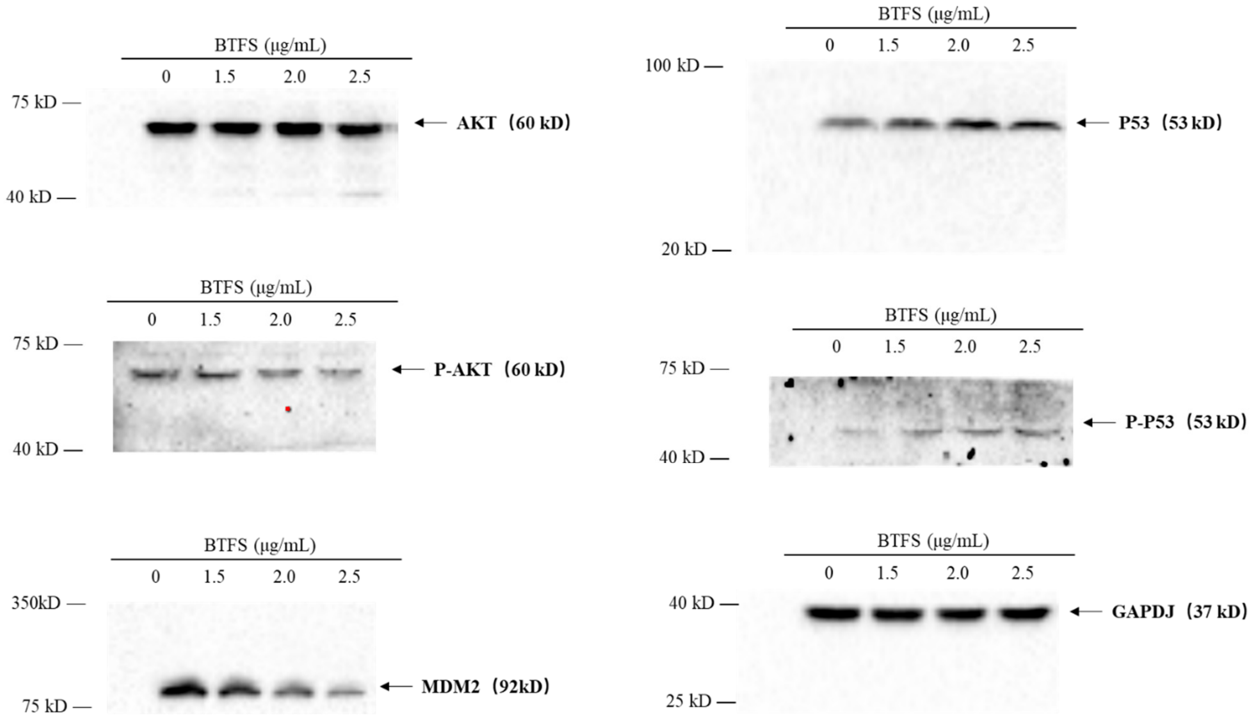

Supplement: Supplementary file 1 [file molecules-25-03515-s001.pdf]
